# The influence of negative mood on heart rate complexity measures and baroreflex sensitivity in healthy subjects

**DOI:** 10.4103/0019-5545.58894

**Published:** 2010

**Authors:** Ralf Köbele, Mandy Koschke, Steffen Schulz, Gerd Wagner, Shravya Yeragani, Chaitra T. Ramachandraiah, Andreas Voss, Vikram K. Yeragani, Karl-Jürgen Bär

**Affiliations:** Department of Psychiatry, Friedrich-Schiller-University, Jena, Germany; 1 Medical Engineering and Biotechnology, University of Applied Sciences, Jena, Germany; 2 Medical School, University of Alberta, Edmonton, Canada, India; 3 Rajiv Gandhi University of Health Sciences, India; 4 Psychiatry and Behavioral Neurosciences, Wayne State University School of Medicine, Detroit, USA and University of Alberta, Edmonton, Canada

**Keywords:** Autonomic nervous system, heart rate variability, major depressive disorder, parasympathetic nervous system, psychophysiology, psychosis, risk of cardiac mortality, vagal

## Abstract

**Background:**

Decreased cardiac vagal function is linked with increased cardiac mortality and depression is associated with decreased heart rate variability. We have previously shown that the Mood Induction Procedure (MIP) in healthy subjects alters pain perception and thalamic activity during pain perception.

**Aim:**

To study the effect of negative emotion on heart rate variability and complexity measures as well as on baroreceptor sensitivity, as these parameters reflect cardiac autonomic function.

**Patients and Methods:**

We studied 20 healthy female controls before and after neutral MIP and 20 healthy female subjects before and after negative MIP. We investigated measures of valence of mood, heart rate variability and complexity and the baroreceptor sensitivity index.

**Results:**

While there was a significant difference in the valence of mood between the neutral and the negative effect condition, there were no significant differences in any of the heart rate or baroreceptor sensitivity measures between the two groups.

**Conclusions:**

Our findings did not show any significant influence of acute negative MIP on heart rate variability and complexity measures and baroreceptor sensitivity, even though depressive disorder and stress are associated with decreased heart rate variability. These findings are discussed in the context of clinical depression and anxiety and the increased risk for cardiac mortality. In contrast to the presented results here, we have previously shown that MIP in healthy subjects alters pain perception and thalamic activity.

## INTRODUCTION

Emotions have diverse effects on autonomic nervous function as illustrated by symptoms such as palpitations and hyperventilation.[1] It is well documented that emotional processes result in changes in heart rate (HR), heart rate variability (HRV) and contractility. Heart rate variability and complexity are useful noninvasive techniques to assess the vagal component of cardiac autonomic function in response to emotional changes. Dishman and colleagues[2] have shown reduced cardiac vagal function among men and women who perceived more stress in the week before the assessment. Moreover, reducing the perceived stress by attending to an emotionally self-management training program increases vagal tone.[3] Similarly, anxiety is often accompanied by somatic manifestations that suggest marked changes in autonomic nervous system (ANS) activity. Reduced HRV has been demonstrated in subjects with various anxiety disorders including patients suffering from panic disorder.[4] This is also true of patients suffering from depression.[5] This is especially important due to the high incidence of sudden unexplained cardiac death in patients with anxiety as well as depression.[6,7] The underlying mechanisms for cardiac vulnerability in depression remain unclear. An altered autonomic neuro-cardiac regulation might be one important pathophysiological factor.[8,9] However, conflicting results were reported in studies that used HRV[10–13] and complexity measures.[5] Baroreflex sensitivity (BRS) and blood pressure variability have also been shown to be decreased in patients with depression,[14,15] thus suggesting decreased parasympathetic function in the disease.

Negative affect is an important symptom of anxiety as well as depression. To elucidate the influence of mood on cardiac autonomic function, we assessed parameters of HRV and complexity in healthy subjects before and after negative mood induction and in a control condition.

## PATIENTS AND METHODS

### Subjects

We investigated 40 female students in this study. Twenty students (age: 24.5 ± 5.1 years) participated in the negative mood induction protocol and the others (age: 25.7 ± 4.3 years) in the control condition. All of them were completely healthy (clinical examination and investigation, ECG, routine blood chemistry; BMI < 26 kg/m^2^), non-smokers and none was receiving medication that would confound the results of this study. The scores on the Hamilton Depression Rating Scale (HAM-D;[16]) and Beck's Depression Inventory (BDI,[17]) were unremarkable.

This study complies with the declaration of Helsinki. All participants gave written informed consent to a protocol approved by the local Ethics Committee of the Medical Faculty of the Friedrich-Schiller University, Jena.

#### Mood induction

To induce sad and neutral emotional states, the modified Velten Mood Induction Procedure (VMIP) was used. The VMIP is amongst the most widely used techniques for studying effective influences upon cognition and behavior,[18] and it has demonstrated effectiveness in altering subjective emotional states.[19] During the VMIP, participants were exposed to a series of 21 self-referent sad-mood statements, which were presented twice and the subjects had to read the statements aloud. While reading the statements, participants were asked to attempt to experience the mood suggested by the statements (e.g. ‘Life is a heavy burden’). Additionally, to facilitate the sad MIP, participants were presented with individually tailored music during VMIP.[20] The procedure is effective and has been used by our group in previous studies.[21]

In contrast, during the neutral procedure, participants were exposed to a series of 21 neutral statements (e.g. ‘An orange is a citrus fruit’), which was similarly presented as the sad statements. Mozart's *Piano concerto No. 21 in C Major* was chosen for all subjects as neutral music. The whole mood-induction procedure lasted approximately 12 min.

To assess effective changes during the experiment, participants were asked to rate the amount of VMIP on the dimensions of valence and arousal using the Self-Assessment Manikin (SAM), an effective rating system devised by Lang.[22] In this system, ratings of valence are indicated by five graphical representations of facial expressions ranging from a severe frown (most sad = − 4) to a broad smile (most positive = +4). For arousal, the Manikin varies from a state of low to high agitation (9 represents a high rating and 1 represents a low rating). The assessment of mood was rated before the mood-induction (test-1), five minutes after induction (test-2) and at the end of the experiment (test-3).

#### Data acquisition and preprocessing

Examinations were performed between 3 and 6 PM and subjects were asked to relax. Respiratory rate was obtained for all patients. The electrocardiogram (high resolution, 1000 Hz) and continuous blood pressure was recorded (CNSystems®, Medizintechnik GmbH, Austria).

#### Heart rate variability

We computed measures of heart rate variability (HRV) in the time and frequency domain.[23] In particular, we obtained the standard deviation of the averages of N-N (normal-to-normal beat) intervals (SDNN, in milliseconds), the square root of the mean squared differences of successive NN intervals (RMSSD). Furthermore, the quotient of low- frequency (LF: 0.04-0.15 Hz) and high-frequency (HF: 0.15-0.4 Hz) powers (LF/HF) was calculated as a measure of relative sympathovagal balance.

#### Compression entropy

This measure calculates the ratio of an original time-series length to a compressed version. An approach to describe the entropy of a text was introduced in the framework of algorithmic information theory.[24] Here, the entropy (complexity) of a given text is defined as the smallest algorithm that is capable of generating the text. In this study, we applied the LZ77 algorithm for loss-less data-compression introduced by Lempel and Ziv.[25] This algorithm is widely used and implemented in many file compressors such as Winzip®. Its application in R-R time-series has been described in detail elsewhere.[9,26] The ratio of the compressed to the original time-series length represents an index of entropy and is referred to as compression entropy (Hc).

#### Symbolic dynamics for high and low variability

For analysis of symbolic dynamics, every single heart beat was compared to the preceding beat. Whenever the beat differed within a specific time limit, this was encoded with the letter ‘0’. In contrast, when beats were different exceeding the given time limits (10 ms), the letter ‘1’ was attributed.[27] From these data, letter sequences were analyzed. For low variability parameters (plvar), the occurrence of sequences containing six consecutive ‘0s were assessed, whereas for high variability parameters (phvar) six consecutive ‘1's were relevant. The time limit applied is always indicated by adding the respective number to the parameter assessed, e.g. plvar10 for low variability with a time limit of 10 ms.

#### Baroreflex sensitivity (b-slope; t-slope)

The baroreflex sensitivity (BRS) was assessed using the sequence method.[28] A detailed description has been published previously.[29] In brief, spontaneous sequences of at least three consecutive beats were analysed, when an increased systolic blood pressure (SBP) of at least 1 mmHg caused an increased BBI (beat-to-beat interval) of at least 5 ms (bradycardic sequence; b-slope) or a decreased SBP caused a decreased BBI (tachycardic sequence; *t*-slope). Furthermore, the numbers of bradycardic and tachycardic baroreflex sequences (b-count; *t*-count) were computed as indicators of baroreflex activation.

#### Nonlinear joint symbolic dynamics (JSDsym; JSDdiam)

**Figure 1:**
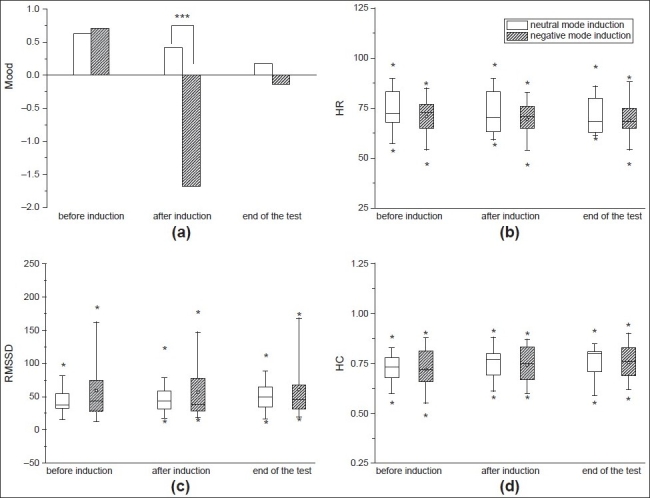
The average dimension of valence was significantly decreased after induction in the negative effect group only (a). The dimension of valence did not differ significantly before induction and at the end of the test (t1, t3). The heart rate for both groups is shown in (b). No significant differences for any condition were observed. Similarly we have not observed a significant difference for RMSSD (c) or compression entropy Hc (d)

To assess heart rate and blood pressure dynamics in a more complex way, an analysis based on joint symbolic dynamics (JSD) was applied,[30] which has been described in detail previously.[31] Here, the beat-to-beat changes of R-R interval and SBP were each coarse-grained to two different symbols: Increasing values are coded as ‘1,’ whereas decreasing and unchanged values were coded as ‘0,’ respectively. Subsequently, short patterns of symbol sequences (words) were formed, and their distribution properties are analysed [probability of symmetric baroreflex-related words (JSDsym), probability of diametric non-baroreflex-related words (JSDdiam), etc.]. Based on the considerations mentioned above, words of a three-letter length are feasible for short-term recordings. [Figure 1]

### Statistical analysis

For statistical analyses, SPSS for Windows (version 14.0) was used. Independent *t*-tests were used to examine differences of dimension of valence and arousal between groups at each point in time (t1, t2, t3).

To compare different effects of both mood-induction procedures on autonomic function, a multivariate analysis of variance for repeated (t1 vs. t2) measures (MANOVA) was performed. In particular, HR and the natural logarithm of RMSSD, HC, sdNN, LF/HF, systolic BP, phvar10, diastolic BP, *t*-slope, b-slope, *t*-count, b-count, JSDsys and JSDdiam were investigated. Follow-up univariate ANOVAs were performed for each parameter. We used Pearson's correlations to explore the bivariate relationship between the questionnaire scores and each physiological parameters (t1, t2, t3). Since groups were matched according to age and sex, no covariates were used.

## RESULTS

### Inferential statistics

There were highly significant differences in mood-valence between the neutral and negative group [Figure 1a] after mood-induction (t2) as shown by an independent *t*-test. (*t* = 9.03, df = 39, *P* > 0.001). As described in Figure 1a, the average dimension of valence was significantly decreased after induction in the negative effect group only, and subjects with sad mood induction reported a significantly decreased sad mood after VMIP. For the dimension of valence before induction and at the end of the test (t1, t3), we found no significant differences between the two groups. We have not found any significant differences for the dimension of arousal.

### Multivariate statistics

Repeated measures MANOVA (t1 vs. t2) for autonomic parameters revealed no significant effect of TIME (Wilks' Lambda = 0.46; *F*(13,11) = 0.98; *P* = 0.52). The analysis also revealed that there was no multivariate difference between both groups (Wilks' Lambda = 0.45, *F*(13,11) = 0.77, *P* = 0.68) or the interaction of TIME × GROUP (Wilks' Lambda = 0.52, *F*(11,24) = 0.18, *P* = 0.1).

### Univariate statistics

Follow-up univariate ANOVAs showed no significant main effects for any variable of the autonomic nervous system (for mean and standard deviation of values, see Table 1 and Figure 1b–d).

**Table 1:** Obtained physiological parameters before and after both mood induction procedures. No significant difference was observed

	Neutral mood induction			Negative mood induction		
				
	Before mood induction	After mood induction		Before mood induction	After mood induction	
sdNN	49.09 ± 46.96	57.25 ± 23.62	n.s.	61.88 ± 62.26	61.86 ± 35.28	n.s.
LF/HF	1.41 ± 1.33	1.40 ± 1.04	n.s.	2.0 ± 1.85	2.0 ± 1.70	n.s.
Phvar10	0.31 ± 0.2	0.32 ± 0.19	n.s.	0.35 ± 0.26	0.33 ± 0.25	n.s.
BPsys	113.86 ± 13.16	111.0 ± 10.9	n.s.	113.56 ± 12.94	113.81 ± 13.35	n.s.
BPdia	72.97 ± 11.76	73.17 ± 9.26	n.s.	72.57 ± 9.06	73.71 ± 9.95	n.s.
b-slope	21.23 ± 8.36	21.15 ± 9.67	n.s.	19.18 ± 9.23	18.70 ± 6.76	n.s.
*t*-slope	19.53 ± 8.14	20.83 ± 10.63	n.s.	18.84 ± 9.25	21.82 ± 9.25	n.s.
JSDsys	0.37 ± 0.14	0.37 ± 0.13	n.s.	0.40 ± 0.11	0.36 ± 0.11	n.s.
JSDdiam	0.03 ± 0.03	0.03 ± 0.03	n.s.	0.02 ± 0.02	0.03 ± 0.03	n.s.
b-count	10.68 ± 7.81	9.50 ± 8.71	n.s.	8.27 ± 6.62	8.50 ± 6.51	n.s.
*t*-count	8.61 ± 8.03	8.82 ± 8.05	n.s.	7.77 ± 7.61	8.23 ± 6.48	n.s.

n.s. = Not significant

### Correlations

There were no significant correlations between the effect scores and the HRV parameters.

## DISCUSSION

Emotions involve a complex mix of cognitive, affective, behavioral and physiological responses.[32] The influence of negative emotions on autonomic cardiac function is important for psychiatric research due to the association of anxiety and depression with increased cardiac mortality.[8,33] Sad mood is a negative affect and in this study, we investigated changes after induction of sad mood in healthy female students and assessed the dimensions of valence and arousal. Significant differences in the dimension of valence are not mirrored in autonomic function, although we performed an analysis applying linear as well as nonlinear parameters of HRV. Besides linear HRV parameters describing the variance of heart beats, nonlinear complexity parameters have been developed to describe the regularity of HR time-series. The application of these novel analyses has led to a higher sensitivity for detecting autonomic dysfunction[5,34] as well as patients at risk for sudden death in different diseases.[35] These results indicate that negative mood per se does not change cardiac autonomic function significantly in healthy subjects as assessed here in the acute condition.[36] This result has important consequences and adds to an ongoing debate on how autonomic function is changed in depression[5] as it might differ between acute versus chronic dysphoric conditions. In addition, decreased BRS has been reported in depression.[14] We did not find any significant changes in BRS during negative VMIP neither by means of linear nor nonlinear parameters for BRS. This probably goes along with our findings of no significant changes in HRV in this study.

One important difference between negative affect in normal controls and patients with clinical depression is one of immediate mood change versus sustained. Our study might indicate that autonomic changes due to negative mood in healthy control subjects are not directly comparable with the depressed mood state in major depression.[5] It is also possible that repeated bouts of negative affect may decrease cardiac vagal function, and thus our acute induction of negative effect is a limitation in our study. The physiological state in depressed patients might be changed due to additional factors, such as hormonal changes in the pituitary gland or even a lack of physical fitness, which probably occurs gradually over the course of time.

On the other hand, duration of the disease and the influence of the disease on other biological systems such as the HPA (hypothalmo-pituitary-adrenal) axis might contribute to autonomic dysfunction.[37] The duration of the disease might be influential since long-term adaptation might occur in depressed patients. The degree of physical fitness is an important issue, as changes of autonomic function in depression might potentially reflect a low degree of physical fitness in such patients. This is of great relevance for cardiac function as well. It is unlikely that the amount of mood induction was not sufficient in our experiment, since we were able to demonstrate significant differences for the dimension of valence and we have found in a previous study differences for pain perception and thalamic activity during pain perception applying the same method.[21] However, the degree of arousal was not different between the neutral and negative condition. It is likely that the degree of arousal is more important than the actual dimension of valence. This degree of arousal should be investigated in future studies and should be applied in psychopathological rating scales used in clinical studies.

## CONCLUSION

In contrast to our previous findings in depressed patients,[5,11] the induced sad mood in healthy subjects in this study revealed opposite findings. Experimentally, induced sad mood in healthy controls highlighted the difference between clinically relevant depressed mood and ‘normal’ variation of sad mood.
